# Life-history responses of insects to water-deficit stress: a case study with the aphid *Sitobion avenae*

**DOI:** 10.1186/s12898-018-0173-0

**Published:** 2018-05-29

**Authors:** Deguang Liu, Peng Dai, Shirong Li, Syed Suhail Ahmed, Zheming Shang, Xiaoqin Shi

**Affiliations:** 1 State Key Laboratory of Crop Stress Biology for Arid Areas, Northwest A&F University, Yangling, Shaanxi Province China; 2 College of Plant Protection, Northwest A&F University, Yangling, Shaanxi Province China

**Keywords:** Drought, Water-deficit stress, Global warming, Life-history traits, Genetic divergence, Adaptation potential

## Abstract

**Background:**

Drought may become one of the greatest challenges for cereal production under future warming scenarios, and its impact on insect pest outbreaks is still controversial. To address this issue, life-history responses of the English grain aphid, *Sitobion avenae* (Fabricius), from three areas of different drought levels were compared under three water treatments.

**Results:**

Significant differences were identified in developmental time, fecundity and adult weight among *S. avenae* clones from moist, semiarid and arid areas under all the three water treatments. Semiarid and arid area clones tended to have higher heritability for test life-history traits than moist area clones. We identified significant selection of water-deficit on the developmental time of 1st instar nymphs and adult weight for both semiarid and arid area clones. The impact of intermediate and severe water-stress on *S. avenae*’s fitness was neutral and negative (e.g., decreased fecundity and weight), respectively. Compared with arid-area clones, moist- and semiarid-area clones showed higher extents of adaptation to the water-deficit level of their respective source environment. Adult weight was identified as a good indicator for *S. avenae*’s adaptation potential under different water-stress conditions. After their exposure to intermediate water-deficit stress for only five generations, adult weight and fecundity tended to decrease for moist- and semiarid-area clones, but increase for arid-area clones.

**Conclusions:**

It is evident from our study that *S. avenae* clones from moist, semiarid and arid areas have diverged under different water-deficit stress, and such divergence could have a genetic basis. The impact of drought on *S. avenae*’s fitness showed a water-level dependent pattern. Clones of *S. avenae* were more likely to become adapted to intermediate water-deficit stress than severe water-deficit stress. After continuous water-deficit stress of only five generations, the adaptation potential of *S. avenae* tended to decrease for moist and semiarid area clones, but increase for arid area clones. The rapid shift of aphids’ life-history traits and adaptation potential under drought could have significant implications for their evolutionary dynamics and outbreak risks in future climate change scenarios.

**Electronic supplementary material:**

The online version of this article (10.1186/s12898-018-0173-0) contains supplementary material, which is available to authorized users.

## Background

Climate change is evident with increasing occurrences of weather extremes like heat waves and dry spells around the globe in recent years according to the report of the Intergovernmental Panel on Climate Change (IPCC) [1]. In China, the annual average atmospheric temperature has increased by at least 1.1 °C over the past several decades till 2007 [2]. The warming trend has been especially evident in northwestern China, where the frequency of dry spells has showed an increase of 19% in the 20th century as compared with the previous couple of centuries (1650–1859) [3].

Increasing frequency and intensity of drought from the global warming trend can have significant impacts on plant growth, morphology and physiology [4]. The changing growth and physiology of plants under drought conditions can in turn have bottom-up effects on the abundances and outbreaks of insect pests, and such effects depend on many factors such as plant type, herbivore species, feeding guild, and stress intensity and duration [5–13]. Drought may also alter the feeding behaviors of herbivorous insects [14, 15], as well as omnivorous insects [16, 17]. Due to their sensitivity to plant water status changes, phloem sap feeders such as aphids are considered as being susceptible to negative impacts of drought conditions, and their strong responses in life-history to drought are often expected [9, 18, 19]. Thus, frequent occurrences of extreme events such as drought in the context of global climate change could alter many biological parameters (e.g., developmental duration and fecundity) and population dynamics of aphids in agricultural and forest ecosystems [20, 21]. For examples, many studies have explored the effects of drought intensity on aphid population growth in terms of 7–10 d fecundity [9, 22, 23]. A few studies have started to focus on changes in developmental durations of nymphs and generation time for aphids under water-deficit stress [20, 24], as well as the effects of continuous drought lasted for multiple aphid generations [21]. However, the impacts of drought on plant-aphid interactions and aphid outbreaks have been hard to predict [4, 20]. Modified plant physiology under drought has been found to have positive, negative or neutral consequences on the performance of aphids [18, 22, 25–27]. Therefore, it remains controversial whether water-deficit stresses can increase aphid outbreaks, even though several hypotheses (e.g., ‘plant stress hypothesis’, ‘plant vigor hypothesis’, and ‘pulsed stress hypothesis’) [21, 28, 29] have been suggested to explain the conflicting results in terms of aphid population dynamics under drought.

Northwestern China provides a good scenario to address this issue. Firstly, drought events have become more frequent and intense in this part of China in the context of the global climate change [30]. Secondly, the cereal aphid, *Sitobion avenae* (Fabricius), is the predominant grain pest in northwestern China [31–33], and increasingly severe damage of this aphid to cereal production seems to be coincident with the warming trend in this part of China [34, 35]. In our previous study, we compared life-history responses under well-watered and moderately water-stressed treatments for *S. avenae* clones from semiarid and moist areas of the Shaanxi Province [20]. Since severe drought incidents are increasingly frequent in northwestern China [20, 30], this study is expanded to include arid areas in both Shaanxi and Gansu Provinces, and a third water treatment (i.e., severe water stress) is incorporated into the experiment. In addition, studies on evolutionary dynamics of plant–insect interactions under relatively long-term water-stress have been rare [19, 20]. Thus, *S. avenae* clones from moist, semiarid and arid areas in northwestern China were collected and tested in the laboratory. We hypothesize that *S. avenae* clones from these areas have differentiated in life-history traits, and the extents of adaptation of these clones to water-deficit conditions can increase after exposure to continuous water-deficit stress for a relatively long period of time. The objectives of our study are to: (1) characterize life-history trait (e.g., developmental duration, fecundity and adult weight) differentiation among these populations under three water-stress treatments; (2) explore the changing pattern of population differentiation after water-stress exposure for five generations of *S. avenae*; and (3) evaluate the adaptation potential for *S. avenae* clones under water-deficit conditions both before and after water-deficit exposure of five generations.

## Results

### Comparison of life-history traits

Population source, water treatment, clone nested in population source, and interactions between the first two factors all showed significant effects on DT5 (the total developmental time of nymphs), 10 d fecundity and adult weight of *S. avenae* clones (Table 1). Population source accounted for 0.8–4.9% of the total variance of the abovementioned traits for generation one, whereas it explained 3.6–10.1% for generation five. The variance from water treatments constituted 5.1–10.7% and 5.5–15.4% of the total variance of each trait for generation one and five, respectively. Interactions between population source and water treatment contributed little to the total variance (generation one: 1.0–3.2%; generation five: 0.8–4.2%). Clone (nested in population source) explained a significant proportion of the total variance for all tested traits (generation one: 36.5–42.9%; generation five: 18.1–36.3%). Clone and population source together accounted for 37.3–47.8% of the total variance for each trait at generation one, whereas they contributed 27.8–39.9% to the total at generation five.

**Table 1:** Estimates of variance components for life-history traits of *Sitobion avenae* populations

Traits	Generation	Variance source	df	*F*	*P*	% total
DT5	1st	Source	2	6.4	*0.002*	*0.8*
		Treatment	2	53.8	*< 0.001*	*6.8*
		Source × treatment	4	11.5	*< 0.001*	*2.9*
		Clone (source)	98	5.9	*< 0.001*	*36.5*
		Error	843	–	–	53.0
	5th	Source	2	65.7	*< 0.001*	*9.7*
		Treatment	2	39.9	*< 0.001*	*5.9*
		Source × treatment	4	14.1	*< 0.001*	*4.2*
		Clone (source)	98	2.5	*< 0.001*	*18.1*
		Error	843	–	–	62.2
10-d Fecundity	1st	Source	2	50.8	*< 0.001*	*4.9*
		Treatment	2	111.8	*< 0.001*	*10.7*
		Source × treatment	4	5.1	*< 0.001*	*1.0*
		Clone (source)	98	9.1	*< 0.001*	*42.9*
		Error	843	–	–	40.5
	5th	Source	2	36.6	*< 0.001*	*3.6*
		Treatment	2	157.3	*< 0.001*	*15.4*
		Source × treatment	4	17.7	*< 0.001*	*3.5*
		Clone (source)	98	7.6	*< 0.001*	*36.3*
		Error	843	–	–	41.3
Adult weight	1st	Source	2	27.4	*< 0.001*	*3.2*
		Treatment	2	43.9	*< 0.001*	*5.1*
		Source × treatment	4	13.8	*< 0.001*	*3.2*
		Clone (source)	98	6.9	*< 0.001*	*39.3*
		Error	843			49.1
	5th	Source	2	77.3	*< 0.001*	*10.1*
		Treatment	2	42.0	*< 0.001*	*5.5*
		Source × treatment	4	3.2	*0.012*	*0.8*
		Clone (source)	98	4.5	*< 0.001*	*28.7*
		Error	843	–	–	55.0

Main effects of population source (source), water stress treatments (treatment), clone nested in source and interactions are shown; DT5, total developmental time of the nymphal stage; significant effects highlighted in italics

**Fig. 1:**
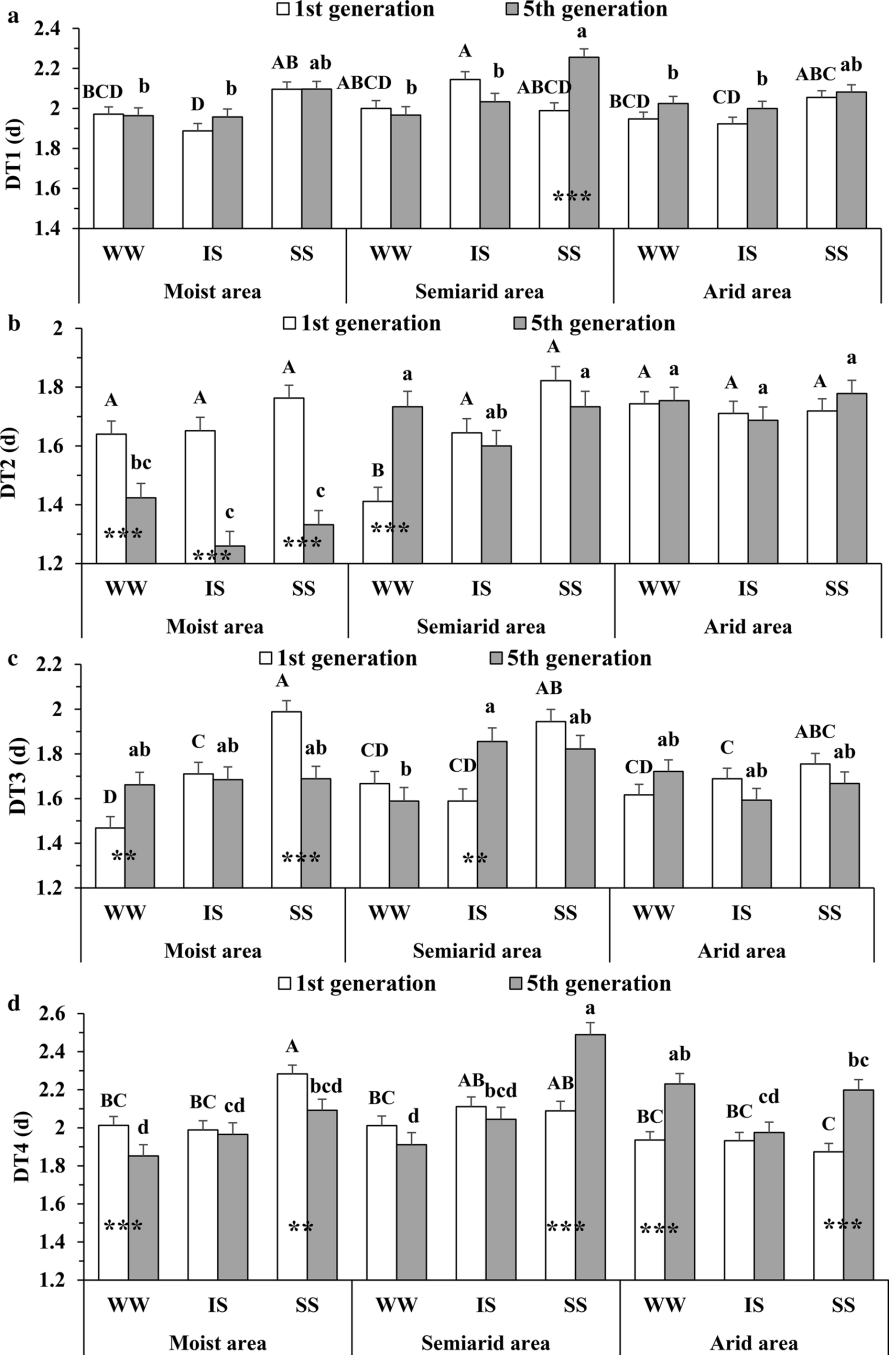
Comparisons of developmental time for 1st and 5th generation *Sitobion avenae* clones under three treatments. DT1 to DT4 (**a**–**d**), the developmental time of 1st to 4th instar nymphs; WW, well-watered treatment; IS, intermediate water deficit; SS, severe water deficit; data with different uppercase and lowercase letters indicate significant differences (α = 0.05, ANOVA followed by Tukey tests) among treatments for generation one and five, respectively; stars in bars indicate significant differences between generation one and five within a treatment for a particular area (*P < 0.05; **P < 0.01; ***P < 0.001)

**Fig. 2:**
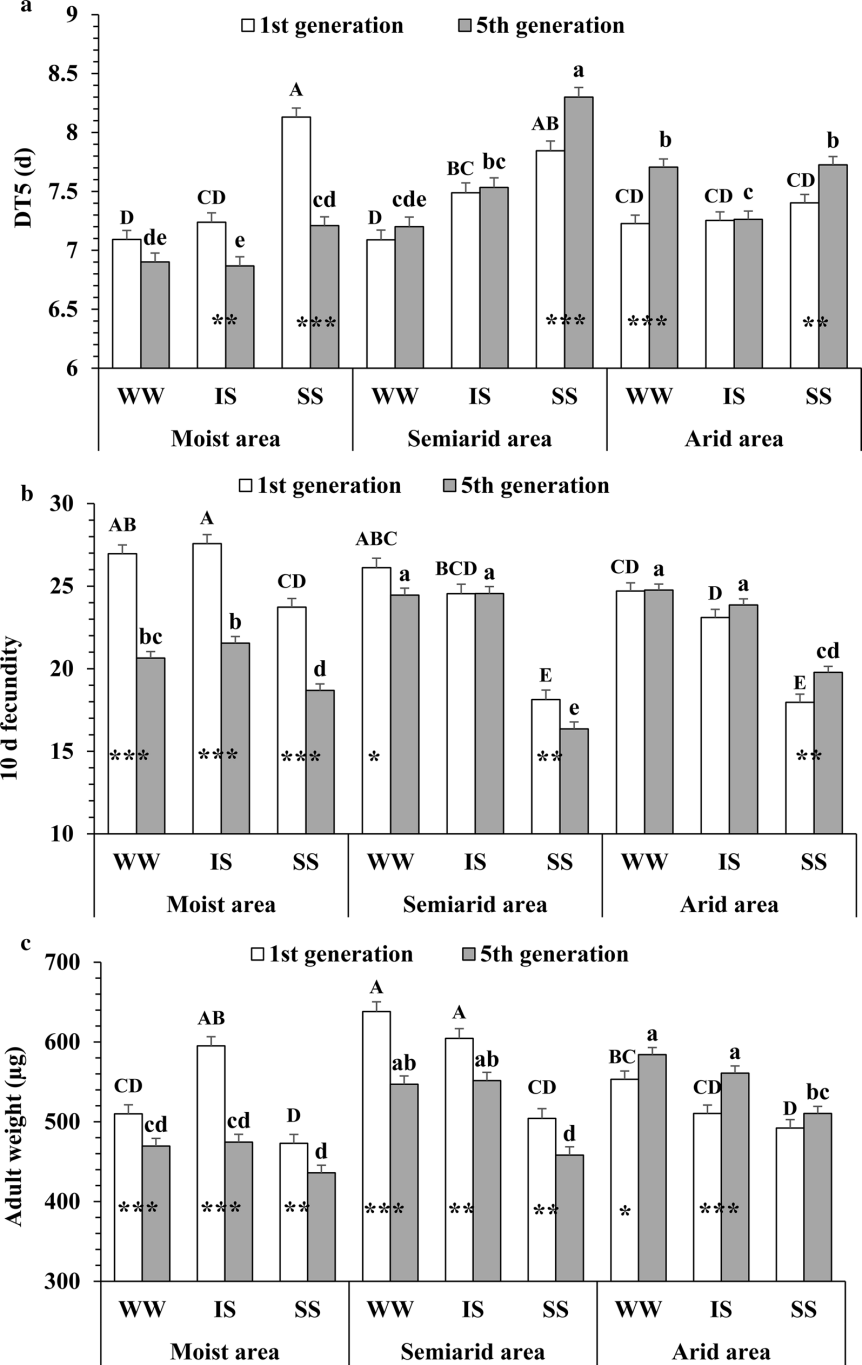
Comparisons of DT5, fecundity and adult weight for different *Sitobion avenae* clones under three treatments. DT5, (**a)**; 10-d fecundity, (**b)**; adult weight, (**c)**; DT5, total duration of the nymphal stage; WW, well-watered treatment; IS, intermediate water deficit; SS, severe water deficit; data with different uppercase and lowercase letters indicate significant differences (α = 0.05, ANOVA followed by Tukey tests) among treatments for generation one and five, respectively; stars in bars indicate significant differences between generation one and five within a treatment for a particular area (*P < 0.05; **P < 0.01; ***P < 0.001)

**Fig. 3:**
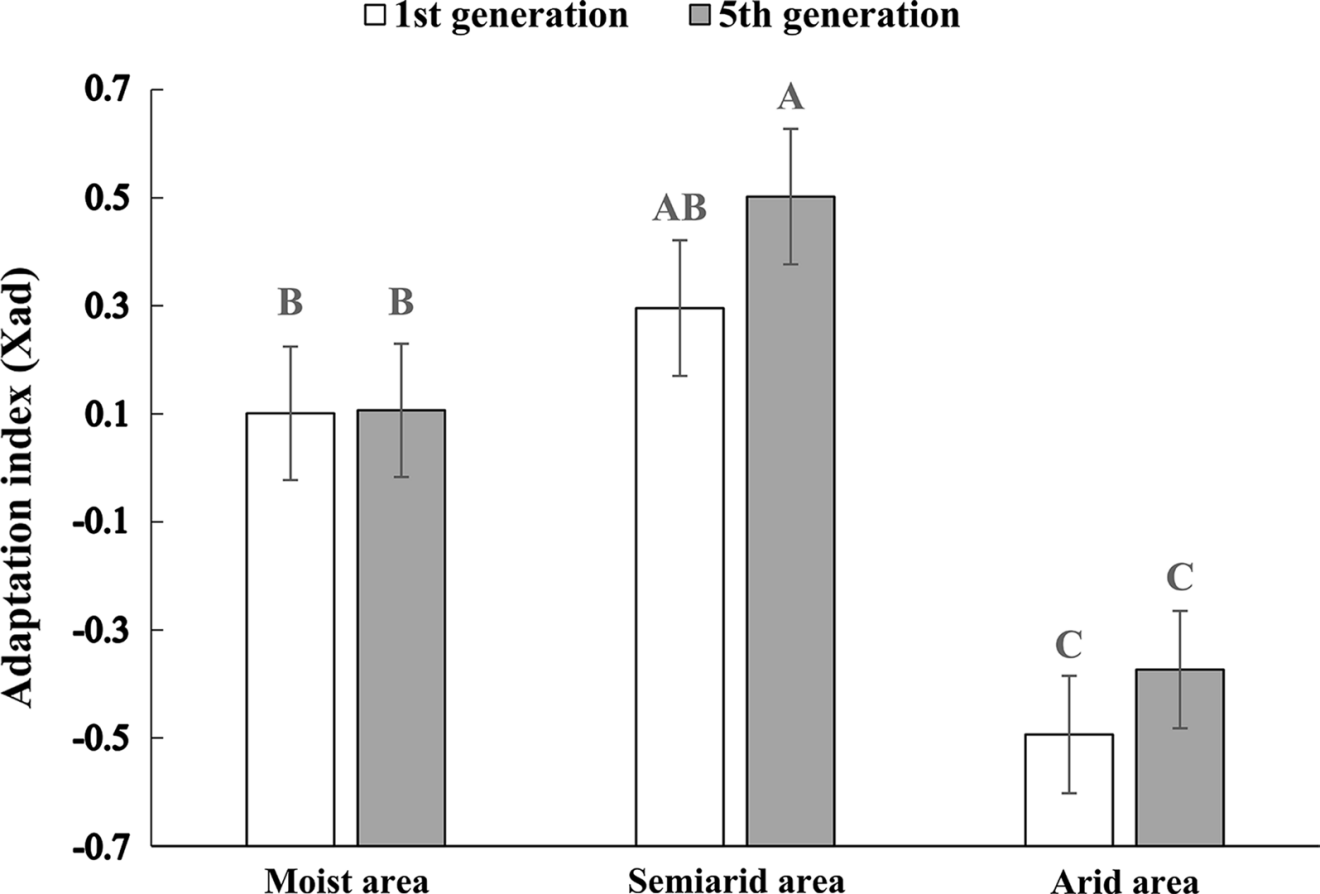
Adaptation indices of 1st and 5th generation clones of *Sitobion avenae* from three areas. X_ad_ > 0, X_ad_ = 0, and X_ad_ < 0 means better, equal, and worse performances, respectively, for *S. avenae* clones under the original water level (e.g., well-watered for moist area clones), compared with those under alternative water levels (e.g., intermediately or severely stressed for moist area clones)

Significant differences in developmental durations of 1st to 4th instar nymphs (DT1 to DT4) and DT5 for *S. avenae* clones were found among their source areas (i.e., moist, semiarid and arid) in many cases, and they tended to be prolonged with increasing water-deficit levels in the source areas: (1) At generation one, semiarid area clones showed a longer DT1 under intermediate water stress than moist area clones (Fig. 1a; *F* = 3.29; df = 2, 843; *P* < 0.05); (2) When tested at generation five, DT2 of moist area clones was shorter under well-watered conditions than that of semiarid or arid area clones (Fig. 1b; *F* = 58.76; df = 2, 843; *P* < 0.001), but this pattern was not found in DT3 (Fig. 1c); (3) At generation five, DT4 of arid area clones was longer under well-watered conditions than that of moist or semiarid area clones (Fig. 1d; *F* = 8.61; df = 2, 843; *P* < 0.001); (4) When tested at generation five, moist area clones had a shorter DT5 under any water-stress treatment than arid area clones (Fig. 2a; *F* = 65.66; df = 2, 843; *P* < 0.001).

The developmental time of each nymphal instar of *S. avenae* showed a tendency to increase under treatments with increasing water-deficit stress: (1) At generation one, semiarid area clones showed a shorter DT2 under well-watered conditions than under both water-deficit treatments (Fig. 1b; *F* = 7.95; df = 2, 843; *P* < 0.001); (2) A longer DT3 was found with increasing water-deficit levels for moist area clones when tested at generation one (Fig. 1c; *F* = 29.57; df = 2, 843; *P* < 0.001); (3) At generation one, moist area clones had a higher DT4 under the severely stressed treatment compared with the well-watered or intermediately stressed treatment (Fig. 1d; *F* = 3.14; df = 2, 843; *P* < 0.05); (4) Moist and semiarid area clones showed a higher DT5 with increasing water deficit levels for both generation one (Fig. 2a; *F* = 53.80; df = 2, 843; *P* < 0.001) and five (*F* = 39.89; df = 2, 843; *P* < 0.001).

Significant differences in the developmental time of *S. avenae* clones were identified after their exposure to intermediate water-deficit stress for five generations. Compared with generation one, DT1 of semiarid area clones was prolonged under severe water stress at generation five (Fig. 1a; *F* = 16.00; df = 1, 149; *P* < 0.001). Similarly, DT3 increased under intermediate water stress for semiarid area clones at generation five (Fig. 1c; *F* = 9.03; df = 1, 149; *P* < 0.01), and DT4 was extended at generation five for semiarid (Fig. 1d; *F* = 19.94; df = 1, 149; *P* < 0.001) and arid (*F* = 22.95; df = 1, 201; *P* < 0.001) area clones under severe water stress. In comparison to generation one, DT5 also increased at generation five for semiarid (Fig. 2a; *F* = 12.01; df = 1, 149; *P* < 0.001) and arid (*F* = 8.34; df = 1, 201; *P* < 0.01) area clones under severe water stress. Thus, the developmental times of *S. avenae* clones tended to increase after their water-deficit exposure for only five generations.

At generation one, moist area clones tended to have a higher 10-d fecundity than semiarid or arid area clones under any of the water treatments (Fig. 2b; *F* = 50.76; df = 2, 843; *P* < 0.001). They showed a lower fecundity than semiarid or arid area clones at generation five under all water treatments except severe water stress (*F* = 36.59; df = 2, 843; *P* < 0.001). Fecundities of moist area clones under well-watered and intermediately stressed conditions were higher than those under severely stressed conditions for both generation one (*F* = 111.84; df = 2, 843; *P* < 0.001) and five (*F* = 157.31; df = 2, 843; *P* < 0.001), and the same patterns were found for semiarid and arid area clones. Compared with generation one, moist and semiarid area clones showed a decrease in fecundity at generation five in all cases except for semiarid area clones under intermediately stressed conditions, whereas arid area clones showed an increase in fecundity under severe water stress at generation five (*F* = 9.79; df = 1, 201; *P* < 0.01).

For both generation one (Fig. 2c; *F* = 27.44; df = 2, 843; *P* < 0.001) and five (*F* = 77.30; df = 2, 843; *P* < 0.001), moist area clones tended to have lower adult weights than semiarid area clones under all water treatments except severe water stress. Little differences in adult weight were found between moist and arid area clones at generation one, but arid area clones had higher adult weights than moist area clones under any water treatment when tested at generation five. At generation one, semiarid area clones tended to have higher adult weights than arid area clones under the three water treatments, but little differences were found between them at generation five. When tested at generation one, moist area clones under intermediate water stress had a higher weight than under severe water stress (*F* = 43.93; df = 2, 843; *P* < 0.001). At both generations, weights of semiarid area clones under the well-watered or intermediately stressed treatment were higher than those under the severely stressed treatment (*F* = 42.01; df = 2, 843; *P* < 0.001). For both generation one and five, arid area clones showed a higher weight under well-watered conditions than under severe water stress. Compared with generation one, moist and semiarid area clones showed a lower weight under all three water stress treatments at generation five (e.g., moist area clones under well-watered conditions, *F* = 11.79; df = 1, 180; *P* < 0.001). However, arid area clones showed a higher weight at generation five than at generation one under well-watered (*F* = 4.19; df = 1, 203; *P* < 0.05) and intermediately stressed (*F* = 14.09; df = 1, 201; *P* < 0.001) treatments.

### Differences in broad-sense heritability of life-history traits

Compared with generation one, the life-history trait heritability of *S. avenae* clones from moist areas increased at generation five for DT1, DT3, fecundity and weight, but decreased for DT2 and DT4 (Table 2). When tested at generation one, semiarid area clones showed significant heritabilities for all tested traits but DT4. Compared with generation one, these clones showed decreased heritabilitiy for DT2 and DT3, but increased heritability for DT4 at generation five. Significant heritabilities for arid area clones were identified in DT1, DT5, fecundity and weight at both generations. Overall, *S. avenae* clones of semiarid and arid areas tended to have more life-history traits with significant heritabilities than those of moist areas. After their exposure to five generations of water-deficit stress, the heritability of certain life-history traits tended to increase for moist area clones, but decrease for semiarid area clones.

**Table 2:** Broad-sense heritability for life-history traits of *Sitobion avenae* clones from moist, semiarid and arid areas

Traits	Moist area clones under WW		Semiarid area clones under IS		Arid area clones under SS	
	G1	G5	G1	G5	G1	G5
DT1	0.333	0.402*	0.467**	0.396*	0.400*	0.426*
DT2	0.500*	0.300	0.571**	0.212	0.333	0.376
DT3	0.200	0.401*	0.452*	0.337	0.403*	0.327
DT4	0.500*	0.189	0.316	0.560**	0.200	0.441*
DT5	0.353	0.356	0.644**	0.571**	0.429*	0.452*
Fecundity	0.014	0.501**	0.592**	0.522**	0.751***	0.745***
Weight	0.018	0.468**	0.738***	0.568**	0.590**	0.455*

*DT1–DT4* the developmental time of 1st to 4th instar nymphs, *DT5* the total developmental time of nymphs, *WW* well-watered treatment, *IS* intermediate water stress, *SS* severe water stress, *G1* generation one, *G5* generation five; the statistical significance of broad-sense heritabilities evaluated with likelihood-ratio tests (LRTs); *P < 0.05; **P < 0.01; ***P < 0.001

### Adaptation index and its correlation with test life-history traits

At generation one, *S. avenae* clones from both moist and semiarid areas showed higher adaptation indices than those from arid areas (Fig. 3; *F *= 26.61; df = 2, 35; *P *< 0.001), but no significant differences in adaptation indices were found between moist and semiarid area clones. At generation five, the adaptation index of moist area clones was lower than that of semiarid area clones, but higher than that of arid area clones. In comparison to generation one, *S. avenae* clones of all the three areas presented no significant changes in adaptation indices at generation five (*F *= 1.28; df = 1, 161; *P *= 0.26).

At generation one, the adaptation indices of moist area clones correlated only with weight among all test life-history traits (Table 3; *r* = 0.7009, *P* < 0.001). When tested at generation five, adaptation indices of these clones correlated to DT4 (*r* = − 0.3623, *P* < 0.05), prin3 (the third factor of PCA) (*r* = − 0.3893, *P* < 0.05) and weight (*r* = 0.4957, *P* < 0.01). At generation one, semiarid area clones showed positive correlations between adaptation indices and weight (*r* = 0.4409, *P* < 0.05), but negative correlations between adaptation indices and prin2 (*r* =  − 0.3680, *P* < 0.05). At generation five, the extent of adaptation for semiarid area clones was negatively correlated to DT3 (*r* = − 0.5710, *P* < 0.001), but it was positively correlated to weight (*r* = 0.6895, *P* < 0.001). The adaptation index for arid area clones was significantly correlated with DT2 (*r* = − 0.3215, *P* < 0.05), DT5 (*r* = − 0.3464, *P* < 0.05), weight (*r* = 0.7850, *P* < 0.001), prin2 (*r* = − 0.3575, *P* < 0.05) and prin3 (*r* = -0.3568, *P* < 0.05) at generation one, but it was only correlated with adult weight at generation five (*r* = 0.6763, *P* < 0.001).

**Table 3:** Correlations between habitat adaptation indices and life-history traits for *Sitobion avenae* clones from three areas

Traits	Moist areas		Semiarid areas		Arid areas	
	G1	G5	G1	G5	G1	G5
DT1	− 0.1865	− 0.0152	− 0.0585	0.2929	− 0.2271	− 0.1717
DT2	− 0.1885	− 0.1076	0.0471	0.2287	− 0.3215*	0.2524
DT3	− 0.1707	0.0489	− 0.2031	-0.5710***	0.0866	− 0.0568
DT4	0.2304	− 0.3623*	0.0892	0.0755	-0.2007	− 0.1650
DT5	− 0.1423	− 0.2500	− 0.0704	-0.0378	-0.3464*	− 0.0874
Adult weight	0.7009***	0.4957**	0.4409*	0.6895***	0.7850***	0.6763***
Prin1	− 0.0134	0.1795	− 0.2606	− 0.1798	0.1584	− 0.0301
Prin2	− 0.0311	0.1020	− 0.3680*	− 0.0156	− 0.3575*	0.0213
Prin3	0.1402	− 0.3893*	− 0.0414	− 0.2761	− 0.3568*	− 0.0850

*DT1–DT4* the developmental time of 1st to 4th instar nymphs, *DT5* the total developmental time of nymphs, *G1* 1st generation, *G5* 5th generation; prin1 to prin3, the first three principal components extracted in PCA; *P < 0.05; **P < 0.01; ***P < 0.001

### Selection coefficients for life-history traits

Under well-watered conditions, moist area clones of *S. avenae* showed significant differentials for DT5 (negative) and weight (positive), and the only significant selection gradient was found for adult weight of these clones at generation five (Table 4). Under intermediate water stress, selection differentials of semiarid area clones were positive for DT1 and adult weight, but negative for DT3 at generation five. The selection gradients for these clones were significantly positive or negative for all traits but DT1. Severe water stress showed relatively little selection for life-history traits of arid area clones at generation five, and significant selection coefficients included the differential of adult weight, and the gradients of DT1 and adult weight.

**Table 4:** Selection differentials and gradients for life-history traits of 5th generation *Sitobion avenae* clones under water-stress

Traits	Moist area clones under WW		Semiarid area clones under IS		Arid area clones under SS	
	Differential	Gradient	Differential	Gradient	Differential	Gradient
DT1	− 0.1153	− 0.0767	0.2413*	0.0492	− 0.1183	− 0.1634*
DT2	− 0.0476	− 0.1061	− 0.0674	− 0.3119***	0.1604	0.1133
DT3	− 0.0215	− 0.0654	− 0.2610*	− 0.2764**	− 0.0233	0.1138
DT4	− 0.1534	− 0.1620	0.0539	− 0.1801*	− 0.0702	0.0271
DT5	− 0.2048*	− 0.1462	− 0.0430	− 0.2228**	− 0.0272	0.0459
Weight	0.4737***	0.4548***	0.6801***	0.7346***	0.6839***	0.6888***

*DT1–DT4* the developmental time of 1st to 4th instar nymphs, *DT5* the total developmental time of nymphs, *WW* well-watered treatment, *IS* intermediate water stress, *SS* severe water stress; *P < 0.05; **P < 0.01; ***P < 0.001

## Discussion

### Divergence and adaptation of *S. avenae* clones

In this study, significant differences in developmental time, 10-d fecundity and adult weight were found among *S. avenae* clones from moist, semiarid and arid areas under any of the three water treatments at generation one of these clones. For examples, at generation one, arid area clones of *S. avenae* had significantly lower fecundities than moist area clones under any of the three water treatments, and they also had lower adult weight than semiarid area clones under well-watered and intermediately stressed conditions. DT1 and DT4, but not DT2 or DT3, tended to be prolonged with increasing water-deficit stress, and this was especially apparent for moist and semiarid area clones. These results indicated that *S. avenae* clones from the three areas had evidently diverged response to water-deficit stress. In addition to 10-d fecundity, various reproductive parameters (e.g., net reproductive rate, lifetime fecundity, and reproductive time) presented significant differences between moist and semiarid area clones of *S. avenae* under intermediate water stress in our previous study [20], providing more evidence of population differentiation between both areas. Although non-genetic environmental effects (e.g., maternal effects and phenotypic plasticity) could contribute to the abovementioned differences in *S. avenae*’s life-history characters, such confounding effects were minimized through rearing all test aphid clones in common laboratory conditions for two to three generations before the initiation of our experiments [36, 37]. Thus, the identified differences among *S. avenae* clones from the three areas with different drought levels could have a genetic basis. Indeed, 18.1–42.9% of the total variation for test life-history characters of *S. avenae* clones was explained by clone (nested in source) alone in the ANOVA, showing substantial genotypic effects. In our previous study, clone also explained an apparently high proportion (i.e., 35.79–83.22%) of the total variance for each life-history trait (e.g., fecundity, reproductive time, and adult lifespan) [20]. In addition, *S. avenae* clones from semiarid and arid areas at generation one tended to have higher heritability (meaning higher possibility of offspring inheritance) for test life-history traits than those from moist areas in this study. This provides another line of evidence that a significant proportion of the life-history differentiation among *S. avenae* populations from areas of different drought levels can be attributed to genetic factors. Thus, genetic divergence among *S. avenae* clones from the three areas could have occurred.

The potential genetic divergence among *S. avenae* clones from moist, semiarid and arid areas also means that these clones may experience substantial selective pressure under drought in the field. Indeed, we found consistently negative selection of intermediate water stress on DT4 for semiarid area clones in the current and previous study [20], and consistently positive selection of well-watered and intermediately stressed conditions on daily fecundity of different *S. avenae* clones from moist and semiarid areas in the previous study [20]. Therefore, it is not unexpected in this study that *S. avenae* clones from moist and semiarid areas showed relatively higher extent of adaptation to the water-stress level of their respective source environment in comparison to those from arid areas. This makes sense since severe water stress in arid areas can cause relatively more extinction events of local aphid clones that obliterate locally adapted gene pools [38], and the extinction-recolonization cycles can be unfavorable to the occurrence of local adaptation for arid area clones. Compared with arid area clones, semiarid area clones presented higher adult weight under intermediate water stress at generation one. This indicates that semiarid area clones should have higher adaptation potential under moderate water stress than arid area clones, since adult weight is shown to be the best indicator of adaptation potential of *S. avenae* clones in this study. Such results add another line of evidence that the occurrence of local adaptation is common for this aphid [34, 37]. The identified changes in adaptation potential of *S. avenae* clones from the three areas at generation one suggest that some genotypes of this aphid might have become adapted to particular drought conditions in the field. Further studies with multiple microsatellites are required to confirm this, especially for those *S. avenae* clones from moist and semiarid areas.

### Effects of relatively longer-term water-deficit exposure

In the present study, *S. avenae* clones of moist, semiarid and arid areas all showed rapid changes in life-history characters after they were kept under intermediate water-deficit stress for only five generations. In comparison to generation one, moist area clones of generation five showed a significant decrease in the total developmental time of nymphs under intermediate and severe water stress, indicating a benefit for these clones after exposure to water-deficit stress for five generations. Despite this benefit, these clones had declined fecundity and adult weight at generation five under any of the water treatments, and the adaptation extent of these clones to well-watered conditions did not change at generation five. This suggests that some of these clones might have adapted to moist conditions in their source area after long-term exposure in the field. Compared with generation one, semiarid area clones of generation five had increased developmental time of nymphs under severe water stress, reduced fecundity under well-watered and severely stressed conditions, and reduced adult weight under all three water treatments, showing significantly negative impact of water-deficit stress that lasted for only five generations. However, the adaptation extent of these clones to intermediate water stress showed no significant changes between generation one and five. This makes sense since these clones may have been subjected to intermediately water-stressed conditions in nature for a long time. Compared with generation one, arid area clones of generation five presented increased fecundity under severe water stress. Similar to *S. avenae* clones of other areas, their experience of intermediate water stress for only five generations was not sufficient to alter the adaptation extent of these clones to severe water stress. although consistent and positive selection of water-deficit stress on adult weight was identified for these clones. Overall, after their exposure to intermediate water-deficit stress for only five generations, adult weight and fecundity tended to decrease for moist- and semiarid-area clones, but increase for arid-area clones, suggesting rapid shifts in vital life-history characters and adaptation potential of this aphid under continuous water-deficit stress for a relatively longer term. This could have significant implications for evolutionary dynamics and outbreak risks of aphids in future climate change scenarios.

### Aphid outbreak risks in the context of global warming

In comparison to well-watered conditions, severe water stress of this study led to decreased fecundity for *S. avenae* clones of all three areas, as well as declined adult weight for all clones except those from moist areas. Therefore, severe water stress could have the potential to lower the abundance and outbreak risk of *S. avenae* populations under future warming scenarios. However, compared with well-watered environments, intermediate water stress showed neutral effects (in terms of fecundity and weight) on *S. avenae* clones in nearly all the cases, and the only exception was that it increased the adult weight of moist area clones at generation one. One explanation for the identified water-level dependent pattern is differential life-history trait plasticity of *S. avenae* clones under variable water-deficit conditions, since phenotypic plasticity of vital characters in this aphid has been shown to evolve as a by-product of adaptation to certain environments [39]. Therefore, the potential risk of *S. avenae* outbreaks can vary depending on the drought level in a particular area under different future warming scenarios.

Predicted climate change can cause increasing intensity and frequency of drought events in many parts of the world, which may have significant consequences for outbreaks of insect pests [1]. Because of their significance in various agricultural systems and sensitivity to water availability [4, 20, 32], aphids’ response to drought has received considerable attention. In some cases, drought has shown little or negative effects on aphids [22–24, 40]. This agrees with our results in this study that severe water stress could lower the adaptability and abundance of *S. avenae* populations. However, populations of quite a few aphid species [e.g., *Diuraphis noxia* (Kurdjumov), *Rhopalosiphum maidis* (Fitch), *Schizaphis graminum* (Rondani)), and *Brevicoryne brassicae* L.] showed enhanced performance and increased outbreaks under drought [41–43]. This is consistent with the finding that the climate change trend appears to have caused increasing outbreaks of cereal aphids (e.g., *S. avenae*) on wheat in China [34, 35]. In this study, we did find that the adaptation potential of arid-area *S. avenae* clones was enhanced after their continuous exposure to intermediate water-deficit stress for only five generations, suggesting a rapid shift of this aphid’s adaptation potential under drought. Based on our current results, we suspect that there are only a small percentage of *S. avenae* genotypes in our samples that have become adapted to particular drought conditions. It is possible that these adapted clones will spread quickly in the future with the increasing pace of the climate warming trend as currently present and less-adapted ones die out. Therefore, we caution against the perception of declining aphid outbreaks under future warming scenarios [44]. There’s an urgent need to determine the number of *S. avenae* genotypes that have become adapted to drought, as well as the pace of their evolution and underlying mechanisms. In addition, other ecological factors (e.g., natural enemies, temperature extremes, CO_2_, aphid genotypes, phenotypic plasticity and secondary endosymbionts) could interact with water-deficit stress to show complicated impacts on aphid outbreaks in the context of global warming [39, 45–47]. For example, the differential responses of *S. avenae* nymphal instars in developmental durations shown in this study could significantly affect parasitism rates under water-deficit stress. Further studies in the abovementioned aspects will make it more practical to predict future outbreaks and changing dynamics of aphids under different climate change scenarios.

## Conclusions

We identified both adaptive and non-adaptive changes in developmental time, fecundity and adult weight among *S. avenae* clones from moist, semiarid and arid areas under all the three water treatments, providing substantial evidence of population divergence under drought for this aphid. Based on analyses of life-history trait variance and heritability, the population divergence of this aphid could have a genetic basis. Clones of *S. avenae* from moist and semiarid areas showed a relatively higher extent of adaptation to the water-stress level of their respective source environment in comparison to those from arid areas. After their exposure to intermediate water-deficit stress for five generations, the fecundity and adult weight of *S. avenae* clones tended to increase for arid areas, but decrease for moist and semiarid areas, indicating that significant shifts in vital life-history characters of this aphid can occur under future warming scenarios. The impact of intermediate and severe water-deficit stress on the fitness *S. avenae* was neutral and negative, respectively. However, we did find that the adaptation potential of arid-area *S. avenae* clones could be enhanced after their continuous exposure to intermediate water-deficit stress for only five generations. Collectively, our data suggest that there should be only low numbers of *S. avenae* genotypes in our samples that have become adapted to particular drought conditions, and these adapted clones could spread quickly in the future with the increasing pace of the climate warming trend. Therefore, prediction of aphid outbreaks under future warming scenarios is much more complicated than expected. Future studies on the number, distribution and evolution of drought-adapted aphid genotypes will provide insight into the genetic structuring and evolutionary ecology of aphid populations under drought, which can make it more practical to predict aphid outbreak risks in the context of global warming.

## Methods

### Aphid collection and colony establishment

**Fig. 4:**
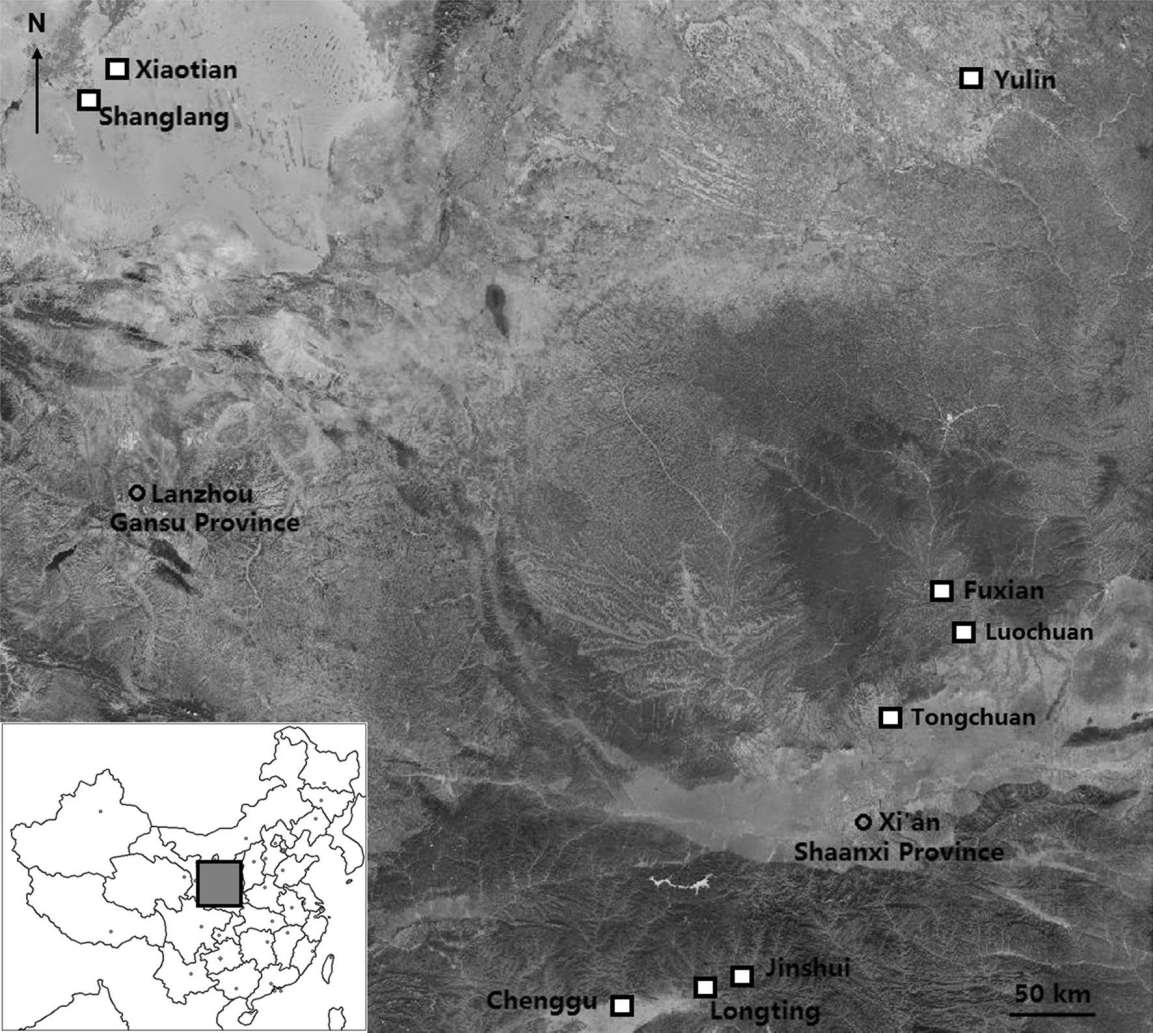
A map of locations for *Sitobion avenae* sampling. Arid area: Yulin Co., 38^o^19′48″ N, 109^o^43′25″ E; Shanglang Town of Mingqin Co., 38°35′48″ N, 103°06′17″ E; Xiaotian Town of of Mingqin Co., 38°36′40″ N, 103°07′18″ E; semiarid area: Yanlian Co., 35^o^ 41′29″ N, 109^o^16′13″ E; Yaozhou Co., 34^o^ 53′38″ N, 108^o^ 58′18″ E; Fuxian Co., 35^o^45′33″ N, 109^o^11′26″ E; moist area: Longting Town of Yangxian Co., 33°12′43″ N, 107°38′30″ E; Jinshui Town of Yangxian Co., 33°16′20″ N, 107°47′45″ E; Chenggu Co., 33^o^07′50″ N, 107^o^16′49″ E

Following Zhao et al. [48] and Bai et al. [30], arid, semiarid, and moist areas were defined as those with a mean annual precipitation up to 200 mm or less, 200–800 mm, and 800 mm or more, respectively. From May to July 2014, aphid populations were collected from three locations for each area (Fig. 4). At least 20 wingless adults (considered as independent clones) were collected from each location. We followed the sampling protocol described in [31] in order to minimize the likelihood of collecting the same clones at a particular location. In the abovementioned three areas*, S. avenae* is usually abundant on cereal crops from April to June of each year. At the three locations in arid areas, the mean trimonthly rainfall of Jan to March, Apr to June, Jul to Sept and Oct to Dec is about 5, 31, 71 and 9 mm, respectively, based on data collected from 1951 to 2008 [49]. The respective trimonthly rainfall in this order is about 21, 95, 240, and 32 mm for semiarid areas, and it is about 47, 252, 501, and 113 mm for moist areas [49]. The collected aphid is not an endangered or protected species, and no special permits have been required for sample collections at all the sites mentioned above.

These collected clones from each location were then reared in segregated colonies. All aphid colonies were established on seedlings (stage 11 to 16 at the Zadoks scale [50]) of wheat (*Triticum aestivum* L. cv. Aikang 58) in 200 ml plastic pots (7 cm in diameter), containing turfy soil mixed with vermiculite and perlite (4:3:1, v/v/v). The wheat cultivar ‘Aikang 58’ is selected for use in our study because of easy manipulations in the laboratory, and it is widely planted in China. Each plant with aphids on it was well covered with a transparent plastic cylinder (6.5 cm in diameter, 15 cm in height, and a 60 mesh net on top). Aphid colonies were maintained in growth chambers under the following conditions: temperature 22 ± 1 °C, relative humidity 65 ± 5%, and photoperiod 16:8 (L:D) h. Prior to the experiment, all test aphid clones were reared on well-watered plants for at least 2–3 generations in the same laboratory conditions mentioned above, since such treatments could minimize confounding environmental effects [37].

After that, aphid clones from each location were randomly selected for use in the following tests. For two of the locations in arid areas (i.e., Yulin and Shanglang), relatively more samples (i.e., over 40) were collected at each location, and 15 clones per location were then selected. For all the other locations, over 20 clones per location were collected in the field, and 10 clones per location were selected. Thus, a total of 100 clones of different areas were used in this study.

### Water-deficit stress treatments

Well-watered and intermediately water-stressed treatments were carried out as described previously in [20]. The severely water-stressed treatment was added in this study, so three water stress treatments were conducted. Each pot of single wheat seedlings (*T. aestivum* cv. Aikang 58) with 35 g (dry weight) of growing substrate in the well-watered, intermediate water-deficit, and severe water-deficit treatments was provided every 3 d by approximately 10, 7, and 5 ml of water, respectively. We used both soil and leaf water potentials to maintain targeted levels of physiological water stress in test plants [51]. Soil moisture was determined with a tensiometer (TEN30, Top Instrument, Hangzhou, China). The soil water potentials for the three treatments were maintained at a range of − 0.02 to − 0.01 MPa, − 0.035 to − 0.02 MPa and lower than − 0.045 MPa, respectively. Water potentials were also measured in plant leaves using the Chardakov method in [52]. The corresponding water potentials of wheat leaves in the three treatments were kept at a range of 0 to − 0.2 MPa, − 0.2 to − 0.6 MPa and − 0.6 to − 0.8 MPa, respectively (for information on dynamics of leaf water potential, see Additional file 1). Targeted water conditions in test pots were maintained by checking soil water potential and the weights of plants with growing media twice a week.

### Life history data collection

The experiment was initially replicated six times per clone, but three replicates were conducted in the late batch of the experiment due to logistic problems. Thus, three or six replicates were conducted for each clone under each treatment. The life-history tests were conducted as detailed previously in [19, 20, 32]. Briefly, each pot of wheat seedlings at the one- to two-leaves stage (11–12 at the Zadoks scale [50]) received one apterous adult of *S. avenae* clones. Wheat seedlings were inspected under room temperatures (about 22 °C) two to three hours later, and all aphid individuals on a test plant were removed except one newborn nymph. The test aphid clones were kept in growth chambers with the abovementioned conditions. They were then monitored until 10 d after the beginning of reproduction for each test aphid individual. Molting, mortality and reproductive events were recorded daily, and the weight of newly molted adults under each treatment was also measured. As detailed previously in [27], developmental durations of 1st, 2nd, 3rd & 4th nymphal instars (referred to as DT1 to DT4 hereafter), total developmental durations of the nymphal stage (referred to as DT5 hereafter), 10-d fecundities (total number of offspring produced in 10 d after the onset of reproduction), and adult weight (for newly emerged adults less than 1 d old) were tabulated. The test plants were replenished every 2 weeks. Using this procedure, the baseline generation-one life-history data for *S. avenae* clones of different areas were recorded. In our preliminary experiments, *S. avenae* clones did not survive under severe water stress after two or three generations, but they survived well under intermediate water stress. Therefore, test *S. avenae* clones of moist, semiarid and arid areas were maintained under intermediate water stress (instead of severe water stress) continuously for five generations. After that, these clones of generation five were subjected to the abovementioned life-history bioassays under three water stress treatments, and their life-history data were then collected.

### Statistical analyses

Three-way nested analyses of variance (nested ANOVA) were used to analyze the abovementioned life-history traits in SAS [53]. We analyzed the fixed effect of ‘population source’ (i.e., moist, semi-arid and arid areas), ‘treatment’ (i.e., well watered, intermediately water-stressed, and severely water-stressed), and their interactions, as well as the random effect of ‘clone’ nested in ‘source’. Treatment means were separated by using Tukey tests following significant ANOVA (α = 0.05). When needed, data were log transformed to meet the assumptions of normality and homoscedasticity in the analyses.

As detailed previously in [36], 10-d fecundity was used as a fitness surrogate in this study. Based on fitness parameters, an index was developed to evaluate extents of plant specialization (or habitat adaptation) for insect clones or populations [36, 54]. Similarly, we can determine the adaptation index (X_ad_) for *S. avenae* clones from different source areas by testing them under water levels of source and alternative environments. If adapted to the source water environment, an aphid clone will have higher fecundity than the average fecundity of the population under the source water level, and it will show higher mean fecundity under the source water level than under alternative water levels. X_ad_ of an aphid clone represents the difference between its fecundities under the source water level and those under alternative water levels. Thus, X_ad_ can reflect the extents of adaptation to the source water environment for the clone involved. X_ad_ values of moist area clones were evaluated by using the following equation (modified from [36, 54]):

$$ X_{ad} = \frac{{\left( {FW - MPFW} \right)}}{MFMP} - \frac{{\left( {FI - MPFI} \right)}}{MFSAP} - \frac{{\left( {FS - MPFS} \right)}}{MFAP} $$

FW, fitness under the well-watered treatment; MPFW, mean population fitness under the well-watered treatment; MFMP, mean fitness of the moist area population; FI, fitness under the intermediate water stress; MPFI, mean population fitness under intermediate water stress; MFSAP, mean fitness of the semiarid area population; FS, fitness under severe water stress; MPFS, mean population fitness under severe water stress; MFAP, mean fitness of the arid area population.

Similarly, X_ad_ values of semiarid and arid area clones of *S. avenae* were respectively determined using:

$$ X_{ad} = \frac{{\left( {FI - MPFI} \right)}}{MFSAP} - \frac{{\left( {FW - MPFW} \right)}}{MFMP} - \frac{{\left( {FS - MPFS} \right)}}{MFAP} $$

and

$$ X_{ad} = \frac{{\left( {FS - MPFS} \right)}}{MFAP} - \frac{{\left( {FI - MPFI} \right)}}{MFSAP} - \frac{{\left( {FW - MPFW} \right)}}{MFMP} $$

The *S. avenae* clones with higher values of X_ad_ should have higher extent of adaptation to the water-deficit level of their source area (i.e., moist, semiarid and arid). The Pearson’s correlations between adaptation indices and life-history traits of *S. avenae* clones were determined by using the PROC CORR procedure in SAS [53]. The principal component analysis (PCA; PROC PRINCOMP in SAS) was conducted with vital life-history traits (including DT1-DT5, and adult weight) after raw data were log-transformed. The factor weightings of each replicate from the PCA were calculated, and they were used as composite life-history factors (i.e., prin1 to prin3) in correlation analyses.

Our life-history tests use clonal aphid lines, and this experimental design allows us to assess the total variance of a particular life-history character (*V*_*P*_), which includes inter-clone components *V*_***G***_ (i.e., the broad-sense genetic variance) and intra-clone components *V*_*E*_ (i.e., environmental variance) [36]. Variance estimates for life-history characters were obtained with the restricted maximum likelihood method by using the software VCE 6.0.2 [55]. Broad-sense heritabilities (*H*^2^ = *V*_*G*_*/V*_*P*_) were then calculated as described previously in [36]. The statistical significance of broad-sense heritabilities was evaluated with likelihood-ratio tests (LRTs) following Carter et al. [56].

In order to evaluate the strength of selection under different test environments (i.e., the water-stressed and well-watered conditions), both differentials and gradients of selection were evaluated by utilizing the PROC REG procedure in SAS as described in detail previously in [32]. Briefly, lifetime fecundity of *S. avenae* female adults was considered as the fitness estimate, and relative fitness of a particular aphid clone was evaluated by dividing the clone’s lifetime fecundity by the average of all clones under each treatment. Standardized selection differentials and gradients were calculated by using simple and multiple linear regressions, respectively (for more details, see [57, 58]).

## Additional file

**Additional file 1.** Dynamics (from day 1 to day 15) of leaf water potential (SE) in wheat seedlings under three water treatments.

## Abbreviations

DT1-DT4: the developmental time of 1^st^ to 4^th^ instar nymphs
DT5: total developmental time of the nymphal stage
WW: well-watered treatment
IS: intermediate water stress
SS: severe water stress
G1: generation one
G5: generation five
X_ad_: adaptation index
