# Supplementary material for: Life-history responses of insects to water-deficit stress: a case study with the aphid Sitobion avenae
Source: BMC Ecol. 2018 May 29;18:17. doi: 10.1186/s12898-018-0173-0 (PMC5975275; doi:10.1186/s12898-018-0173-0)
Supplement: Supplementary file 1 — Additional file 1. Dynamics (from day 1 to day 15) of leaf water potential (SE) in wheat seedlings under three water treatments. [file 12898_2018_173_MOESM1_ESM.pdf]

Additional File 1. Dynamics (from day 1 to day 15) of leaf water potential (SE) in wheat seedlings under three water treatments

Day	Treatment		
	Well-watered (MPa)	Intermediately stressed (MPa)	Severely stressed (MPa)
1	-0.1583 (0.0130)	-0.2417 (0.0154)	-0.6007 (0.0217)
2	-0.1790 (0.0106)	-0.3483 (0.0083)	-0.6517 (0.0201)
3	-0.1950 (0.0112)	-0.4250 (0.0050)	-0.6959 (0.0356)
4	-0.1450 (0.0157)	-0.2750 (0.0112)	-0.6067 (0.0238)
5	-0.1667 (0.0105)	-0.4017 (0.0210)	-0.6600 (0.0224)
6	-0.1917 (0.0154)	-0.4600 (0.0126)	-0.6903 (0.0211)
7	-0.1317 (0.0117)	-0.3350 (0.0157)	-0.6100 (0.0321)
8	-0.1708 (0.0067)	-0.3933 (0.0148)	-0.6633 (0.0307)
9	-0.1917 (0.0164)	-0.4700 (0.0134)	-0.7009 (0.0347)
10	-0.1433 (0.0141)	-0.4150 (0.0285)	-0.6033 (0.0343)
11	-0.1687 (0.0067)	-0.4483 (0.0197)	-0.6402 (0.0211)
12	-0.1933 (0.0167)	-0.4683 (0.0220)	-0.7099 (0.0365)
13	-0.1501 (0.0122)	-0.3950 (0.0207)	-0.6178 (0.0229)
14	-0.1759 (0.0103)	-0.4508 (0.0197)	-0.6817 (0.0241)
15	-0.1985 (0.0115)	-0.4895 (0.0239)	-0.7210 (0.0309)

Note: The experiment was initiated 5 to 7 d after planting.
